# Alveolar Periostitus

**Published:** 1865

**Authors:** H. A. Smith


### DENTAL REGISTER OF THE WEST

VOL. XIX.] MAY, 1865. [No. 5.

### Original Essays and Communications.

### ALVEOLAR PERIOSTITUS.

BY H. A SMITH.

Read before the Mississippi Valley Dental Association.

Alveolar Periostitus, as the name implies, involves the lining membranes of the sockets of the teeth.

It is only by a close observation of the manifestations of a diseased condition like *Dental Periostitus*, that we can hope to obtain a clear insight into its nature. And this presupposes a knowledge of the anatomy and physiological functions of the parts involved.

Although it is generally agreed that there is but one tissue connecting the fang of the tooth with the alveolus, still there are some reasons to believe there are *two*. Mr. Spencer Bates, of England, describes two,—the *peridenteum* of of the tooth and the *periostium* of the bone. The first he regards as similar to the tissue of the skin, the other like the periostium of other bones.

One of the reasons for assuming the presence of two membranes differing thus in structure is, that we never have osseous union of the tooth with the jaw. In other parts anchylosis is frequently the result of inflammation in the periostial membrane. The absence of this result, it is claimed, can only be accounted for by the presence of these two tissues differing thus widely in their structure. Until it can be demonstrated, however, that two membranes are present, we will assume that there is but one, viz: the periosteum. This membrane, similar to the periosteum of other bones, is described as a delicate fibrous tissue, highly vascular and readily takes on inflammatory conditions.

The *causes* that produce periostitus are various; but in a majority of cases, perhaps, coming under our notice, it is the result of inflammation in the pulp of the tooth, either from direct exposure, or the too near proximity of some irritating substance, as a metal filling for instance, thus subjecting the pulp to variations in temperature. Whatever may be the cause, the symptoms attending the disease are generally the same. At first there is a slight sensation of uneasiness, and a feeling of fullness, with a desire to press the teeth together or move with the finger the teeth involved, and unless arrested the pain increases, becoming dull, heavy and continuous. These symptoms are soon followed by physical signs; the gums become of a reddish or purple color, and are often swollen and very tender. At first the inflammation is confined about the neck of the tooth involved, but as the pain increases, a more general inflammation of the gums usually follows.

At this stage suppuration will set in and pus is secreted at or around the end of the fang. The pus may find its way out through the alveolus, and we have then a well defined *alveolar* abscess,—or if the whole of the periosteum takes on the inflammatory condition, and the tooth is not firmly set in the jaw, the periosteum will become detached from the neck of the tooth and the secretion will escape at the margin of the gums.

If the inflammation begins at the margin of the gum and extends downwards it is not usually attended with so much pain as when it commences in the pulp and afterwards attacks the periosteum at the apex of the fang, for the reason that in the first instance the effete matter finds a more easy escape. It is in those cases where the tooth is firmly set in a thick heavy alveolus, that the pain experienced is most severe; as in a case of rheumatism, when it attacks the joints the inflammation is most violent and painful, because of the density of the tissues involved, and the unyielding nature of the tendons and faciæ which bind them together.

In young subjects, and also those in which the connection of the tooth with the socket is slight, comparatively, the tooth is more easily raised in the alveolus, thus giving room for the effused matter. The tension upon the vessels is, as a result, relieved, and consequently we find little pain. This class of cases do not so quickly terminate in abscess as in others described.

It was stated that in a large proportion of cases of periostitus they resulted from inflamation first in the pulp of the tooth extending through the foramen and upwards, involving a portion or all of the lining membrane of the sockets. In these cases if suppuration is established, the death of the pulp is nearly certain, as well as necrosis of the tooth itself, if the periosteum become separated from the cementum, throughout the entire socket. But in those cases caused by the accumulation of tartar or other irritants about the neck of the tooth, and beginning at the margin of the gum extending downwards, we do not so certainly find the vitality of the pulp destroyed. The periosteum may by the suppurative process be nearly or quite destroyed to the apex of the fang, and yet the tooth retain its vitality through the pulp.

We have attempted a description of the symptoms of alveolar periostitus, together with some of the causes that may induce them; but frequently cases present, attended by the usual symptoms of periostitus, and yet they seem not to be amenable to the usual treatment in such cases; neither do they terminate in suppuration and abscess. The patient suffers long past the period when we should expect either a cure or a termination of the inflammation in the usual manner.

The cause of this condition is obscure, but it may perhaps be due to the fact that the fang of the tooth has become exostosed. If we extract a tooth in which these conditions are present, we shall find upon examining the periosteum streaks or patches of a reddish color scattered over its surface. These deposits are often of considerable thickness and size. As these deposits of lymph, or whatever they may be, become more dense calcification takes place and we have a deposit of bone. The suffering in these cases depends upon the slowness or rapidity of the growth of this deposit. If formed too rapidly for the necessary absorption of the alveolus to accommodate it, the pain would be very acute, unless the tooth could rise in its socket, and the pressure be relieved in that manner. What in the first diagnosis of the case was pronounced periostitus has now passed to a variety of exostosis.

It is perhaps for the reason that exostosis is *usually* unattended with any considerable suffering, that we fail to recognize this condition until the tooth is extracted.

Periostitus cannot attack a tooth very frequently without terminating in suppuration and abscess. And if we have a succession of these attacks without the usual results, it is more than probable that the tooth will be lost from pain induced by exostosis of the fang.

